# Supplementary material for: Protecting young children from future pandemics: getting the basics right
Source: BMJ Paediatr Open. 2023 Jun 1;7(1):e002008. doi: 10.1136/bmjpo-2023-002008 (PMC10254943; doi:10.1136/bmjpo-2023-002008)
Supplement: Author's manuscript [file bmjpo-2023-002008.draft_revisions.pdf]

BMJ Paediatrics Open

BMJ Paediatrics Open is committed to open peer review. As part of this commitment we make the peer review history of every article we publish publicly available.

When an article is published we post the peer reviewers' comments and the authors' responses online. We also post the versions of the paper that were used during peer review. These are the versions that the peer review comments apply to.

The versions of the paper that follow are the versions that were submitted during the peer review process. They are not the versions of record or the final published versions. They should not be cited or distributed as the published version of this manuscript.

BMJ Paediatrics Open is an open access journal and the full, final, typeset and author-corrected version of record of the manuscript is available on our site with no access controls, subscription charges or pay-per-view fees (http://bmjpaedsopen.bmj.com).

If you have any questions on BMJ Paediatrics Open's open peer review process please email info.bmjpo@bmj.com

BMJ Paediatrics Open

Protecting young children from future pandemics: Getting the basics right

Journal:	*BMJ Paediatrics Open*
Manuscript ID	bmjpo-2023-002008
Article Type:	Viewpoint
Date Submitted by the Author:	04-Apr-2023
Complete List of Authors:	Lu, Chunling; Brigham and Women's Hospital, ; Harvard Medical School, Department of Global Health and Social Medicine<br>Behrman, Jere; University of Pennsylvania, Department of Economics<br>Richter, Linda; University of the Witwatersrand Johannesburg, DSI-NRF Centre of Excellence in Human Development
Keywords:	COVID-19, Growth

SCHOLARONE™
Manuscripts

I, the Submitting Author has the right to grant and does grant on behalf of all authors of the Work (as defined in the below author licence), an exclusive licence and/or a non-exclusive licence for contributions from authors who are: i) UK Crown employees; ii) where BMJ has agreed a CC-BY licence shall apply, and/or iii) in accordance with the terms applicable for US Federal Government officers or employees acting as part of their official duties; on a worldwide, perpetual, irrevocable, royalty-free basis to BMJ Publishing Group Ltd ("BMJ") its licensees and where the relevant Journal is co-owned by BMJ to the co-owners of the Journal, to publish the Work in this journal and any other BMJ products and to exploit all rights, as set out in our licence.

The Submitting Author accepts and understands that any supply made under these terms is made by BMJ to the Submitting Author unless you are acting as an employee on behalf of your employer or a postgraduate student of an affiliated institution which is paying any applicable article publishing charge ("APC") for Open Access articles. Where the Submitting Author wishes to make the Work available on an Open Access basis (and intends to pay the relevant APC), the terms of reuse of such Open Access shall be governed by a Creative Commons licence – details of these licences and which Creative Commons licence will apply to this Work are set out in our licence referred to above.

Other than as permitted in any relevant BMJ Author's Self Archiving Policies, I confirm this Work has not been accepted for publication elsewhere, is not being considered for publication elsewhere and does not duplicate material already published. I confirm all authors consent to publication of this Work and authorise the granting of this licence.

Confidential: For Review Only

Protecting young children from future pandemics: Getting the basics right

Since the outbreak of COVID-19, a number of global initiatives have been established, primarily aimed at coordinating global-, regional-, and country-level efforts to develop, produce, distribute and improving uptake of biomedical technologies to fight the pandemic.[1] The goal is to stop the pandemic and rapid recover economic and societal activities by accelerating development of vaccines, therapeutics, and diagnostics and supporting, especially low- and middle-income countries (LMICs), in translating newly developed tools into national responses to the pandemic. Efforts and achievements of global partnerships for combating COVID-19 are applauded as the "world's most comprehensive end-to-end solution".[1] The models used for coordinating actions between unprecedentedly large numbers of stakeholders offer invaluable lessons and experiences for preparation for future pandemics.

Despite these exciting achievements, it is important to note that global activities have focused mainly on enhancing biomedical capacity for disease control; little attention has been given to improving the social conditions, i.e., getting millions of households ready for future pandemics. For pandemics such as COVID-19, as for most health challenges, social conditions are very important, in some cases more important than biomedical capacities for disease control.

We argue that developing and distributing new medical technologies is necessary, but not sufficient for conquering the future pandemics. As recommended by the World Health Organization,[2] for viral infections caused by respiratory droplets, physical contacts, and contaminated food or water, ensuring household have basic sanitation, hygiene facilities, clean water, ability to quarantine, and access to information is crucial for effective prevention and safe care at home. Strengthening household living conditions and their socioeconomic infrastructure should be included in global preparation as an essential component, especially given the lag between discovery and mapping of a disease and invention or adaptation of tools for controlling it. Developing new technologies requires knowledge about the virus and long-time scientific research on robust methods of delivery. The success of developing the mRNA COVID-19 vaccine within a short time built upon decades of rigorous scientific investigations; nevertheless, the lag from virus discovery to the first vaccine delivery was a year.[3] Before those tools became available, we were reliant on prevention methods, such as home quarantine and hygiene practices, to limit viral spread. Additionally, even after vaccines or treatments are developed, ensuring equal access to these tools for populations in need, especially those in LMICs, has proven to be very difficult due to technical, operational, financial, and informational challenges. Even in countries with high rates of vaccination, home quarantine, handwashing, physical distancing, and masks are still required to curb transmission. Empowering households with the means for preventing the spread of a pandemic remains pivotal to the success of reducing prevalence and mortality.

Empirical evidence about household preparedness for COVID-19 in LMICs is worrying. In our investigation of 56 LMICs with available nationally representative data,[4] on average <20% of young children lived in households that meet five preparedness conditions for preventing COVID-19 or communicable diseases: space for quarantine (defined by three persons per sleeping room), basic sanitation, soap and water for handwashing, phone for communication, and exposure to mass media. Children in low-income countries or sub-Saharan Africa were the most disadvantaged – only 4.4 % or 4.6%, respectively, lived in prepared households. In upper-

https://mc.manuscriptcentral.com/bmjpo

Confidential: For Review Only

middle-income countries, such as Paraguay, a large and significant disparity in COVID-19 preparedness was found between the poorest households (10.7%) and the richest (90.2%). Lacking access to basic sanitation and/or hygiene facilities are the two main contributors to poor household preparation in the 56 countries. These findings suggest that home care or confinement is currently beyond the capacity of many low-income households, potentially compounding a range of health risks to young children when they were locked down at home by government regulations.

Progress in improving household physical conditions has been very slow. In sub-Saharan Africa, the average population coverage of basic sanitation increased from 23% in 2000 to 30% in 2017, and only 25% lived in households with handwashing facilities in 2017.[5] The importance of these services in protecting households from infections and the slow progress in coverage requires immediate attention and action from governments, global health and financial organizations, donors, and other stakeholders. Improving household preparedness for future pandemics should be included in global preparation strategies. More political commitments and financial investments should be made to empower low-income households in preparing for future pandemics by improving their access to basic sanitation, hygiene, housing, information, and communication tools.

We declare no competing interests.

Funding sources: UKRI GCRF Collective Fund Award (Grant Ref: ES/T003936/1) to the University of Oxford, UKRI ECRS GCRF, Harnessing the power of global data to support young children's learning and development: Analyses, dissemination and implementation.

References

1. Moon, S., Armstrong, J., Hutler, B., Upshur, R., Katz, R., Atuire, C., ... & Wolff, J. (2021). Governing the access to COVID-19 tools accelerator: towards greater participation, transparency, and accountability. *The Lancet*.
2. World Health Organization. Coronavirus disease (COVID-19): Home care for families and caregivers. https://www.who.int/news-room/q-a-detail/coronavirus-disease-covid-19-home-care-for-families-and-caregivers. 2020 (Geneva: World Health Organization). Accessed 3 May 2021.
3. United States Central for Disease Control and Prevention (CDC). CDC Museum COVID-19 Timeline. https://www.cdc.gov/museum/timeline/covid19.html#:~:text=December%2012%2C%202019,shortness%20of%20breath%20and%20fever (accessed June 6, 2022)
4. Lu C, Luan Y, Naicker SN, Subramanian SV, Behrman JR, Heymann J, Stein A, Richter LM. Assessing the prevalence of young children living in households prepared for COVID-19 in 56 low- and middle-income countries. Glob Health Res Policy. 2022 Jun 21;7(1):18. doi: 10.1186/s41256-022-00254-2. PMID: 35729611; PMCID: PMC9210057.
5. Progress on household drinking water, sanitation and hygiene 2000-2017. Special focus on inequalities. New York: United Nations Children's Fund (UNICEF) and World Health Organization, 2019. https://www.unicef.org/reports/progress-on-drinking-water-sanitation-and-hygiene-2019

Confidential: For Review Only

BMJ Paediatrics Open

Protecting young children from future pandemics: Getting the basics right

Journal:	*BMJ Paediatrics Open*
Manuscript ID	bmjpo-2023-002008.R1
Article Type:	Viewpoint
Date Submitted by the Author:	04-May-2023
Complete List of Authors:	Lu, Chunling; Brigham and Women's Hospital, ; Harvard Medical School, Department of Global Health and Social Medicine<br>Behrman, Jere; University of Pennsylvania, Department of Economics<br>Richter, Linda; University of the Witwatersrand Johannesburg, DSI-NRF Centre of Excellence in Human Development
Keywords:	COVID-19, Growth

SCHOLARONE™
Manuscripts

I, the Submitting Author has the right to grant and does grant on behalf of all authors of the Work (as defined in the below author licence), an exclusive licence and/or a non-exclusive licence for contributions from authors who are: i) UK Crown employees; ii) where BMJ has agreed a CC-BY licence shall apply, and/or iii) in accordance with the terms applicable for US Federal Government officers or employees acting as part of their official duties; on a worldwide, perpetual, irrevocable, royalty-free basis to BMJ Publishing Group Ltd ("BMJ") its licensees and where the relevant Journal is co-owned by BMJ to the co-owners of the Journal, to publish the Work in this journal and any other BMJ products and to exploit all rights, as set out in our licence.

The Submitting Author accepts and understands that any supply made under these terms is made by BMJ to the Submitting Author unless you are acting as an employee on behalf of your employer or a postgraduate student of an affiliated institution which is paying any applicable article publishing charge ("APC") for Open Access articles. Where the Submitting Author wishes to make the Work available on an Open Access basis (and intends to pay the relevant APC), the terms of reuse of such Open Access shall be governed by a Creative Commons licence – details of these licences and which Creative Commons licence will apply to this Work are set out in our licence referred to above.

Other than as permitted in any relevant BMJ Author's Self Archiving Policies, I confirm this Work has not been accepted for publication elsewhere, is not being considered for publication elsewhere and does not duplicate material already published. I confirm all authors consent to publication of this Work and authorise the granting of this licence.

Confidential: For Review Only

Protecting young children from future pandemics: Getting the basics right

The United Nations Convention on the rights of the child has defined four basic rights of children: to survival, to protection, to development, and to participation.[1] Protecting young children from the direct and secondary impacts of the COVID-19 and future pandemics and ensuring their survival and healthy development requires progress in both technology and household socioeconomic infrastructure.

Since the outbreak of COVID-19, a number of global initiatives have been established, primarily aimed at coordinating global-, regional-, and country-level efforts to develop, produce, distribute and improving uptake of biomedical technologies to fight the pandemic.[2] The goal is to stop the pandemic and rapidly recover economic and societal activities by accelerating the development of vaccines, therapeutics, and diagnostics and supporting, especially low- and middle-income countries (LMICs), in translating newly developed tools into national responses to the pandemic. Efforts and achievements of global partnerships for combating COVID-19 are applauded as the "world's most comprehensive end-to-end solution".[2] The models used for coordinating actions between unprecedentedly large numbers of stakeholders offer invaluable lessons and experiences for preparation for future pandemics.

Despite these exciting achievements, it is important to note that global activities have focused mainly on enhancing biomedical capacity for disease control; little attention has been given to improving social conditions by helping millions of households to be better prepared for future pandemics. Social conditions are very important for pandemics such as COVID-19, as for most health challenges; in some cases more important than biomedical capacities for disease control.

We argue that developing and distributing new medical technologies is necessary, but not sufficient for conquering future pandemics. As recommended by the World Health Organization,[3] for viral infections caused by respiratory droplets, physical contacts, and contaminated food or water, ensuring households have basic sanitation, hygiene facilities, clean water, ability to quarantine, and access to information is crucial for effective prevention and safe care at home. Strengthening household living conditions and their socioeconomic infrastructure should be included in global preparation as an essential component, especially given the lag between discovery and mapping of a disease, and invention or adaptation of tools for controlling it.

Developing new technologies requires knowledge about the virus and long-time scientific research on robust methods of delivery. The success of developing the mRNA COVID-19 vaccine within a short time built upon decades of rigorous scientific investigations; nevertheless, the lag from virus discovery to the first vaccine delivery was a year.[4] Before those tools became available, we were reliant on prevention methods, such as home quarantine and hygiene practices, to limit viral spread. Additionally, even after vaccines or treatments are developed, ensuring equal access to these tools for populations in need, especially those in LMICs, has proven to be very difficult due to technical, operational, financial, and informational challenges. Even in countries with high rates of vaccination, home quarantine, handwashing, physical distancing, and masks are still required to curb transmission. Empowering households with the means for preventing the spread of a pandemic remains pivotal to the success of reducing prevalence and mortality.

Confidential: For Review Only

Empirical evidence about young children living in households with preparedness for COVID-19 in LMICs is worrying. In our investigation of 56 LMICs with available nationally representative data,[5] on average <20% of young children lived in households that meet five preparedness conditions for preventing COVID-19 or communicable diseases (Panel 1). Children in low-income countries or sub-Saharan Africa were the most disadvantaged – only 4.4 % or 4.6%, respectively, lived in prepared households. In most countries, significant residence- and/or wealth-disparities in household preparedness were observed, favoring children living in urban areas or in the richest households. For example, in Paraguay, the gap in young children living in prepared households between the poorest (10.7%) and the richest (90.2%) was close to 80 percentage points. Lacking access to basic sanitation and/or hygiene facilities are the two main contributors to poor household preparation in the 56 countries. Evidence in high-income countries showed that minorities living in overcrowded households with poor hygiene had higher transmission risk of COVID-19 than their counterparts.[6] These findings suggest that home care or confinement is currently beyond the capacity of many low-income households, potentially compounding a range of health risks to young children when they were locked down at home by government regulations.

Ensuring everyone (including children) having access to clean water, save basic sanitation, and hygiene by 2030 is one of the Sustainable Development Goals (SDG 6),[7] though progress in achieving SDG 6 in low-income settings has been very slow. In sub-Saharan Africa, the average population coverage of basic sanitation increased from 23% in 2000 to 30% in 2017, and only 25% lived in households with handwashing facilities in 2017.[7] As extreme weather becomes more frequent due to the climate change, delivering WASH services is more challenging in low-income countries. The importance of these services in protecting households from infections and the slow progress and increasing challenges in coverage requires immediate attention and action from governments, global health and financial organizations, donors, and other stakeholders.

In many LMICs, current investments in water, sanitation, and hygiene (WASH) are far from sufficient for achieving SDG 6. Low-income countries have limited domestic tax revenue for implementing their plans on WASH due to their low economic development as well as revenue leaks, including global tax abuse facilitated by high-income countries in some respects. It has been estimated that increasing governmental revenues equivalent to global tab tax abuse could be associated with 36 million more people having access to basic sanitation and 18 million more to basic drinking water in LMICs.[8] However, aid for WASH in LMICs has experienced a fall since the SDGs era (by 5.6% between 2017 and 2020).[9] To prepare households in LMICs for future pandemics, high-income countries should increase their aid for WASH, providing compensation to LMICs for climate damages caused by global warming, and help LMICs achieve their targets on SDG 6.

To summarize, ensuring children's basic rights during the pandemics requires improvement of their living environment. Getting low-income households prepared for future pandemics should be part of the global preparation strategies and deserves more political commitments and financial investments.

Confidential: For Review Only

Panel 1 Household preparedness for the COVID-19 and future pandemics[4]

1. Access to basic hygienic facilities: measured by availability of handwashing facilities with water and soap
2. Access to basic sanitation facilities: measured by access to sanitation not shared with other households and with hygienic separation of excreta from human contact (e.g., flush to piped sewer system/septic tank/pit latrine, ventilated improved pit, pit latrine with slab, etc.)
3. Adequate quarantine conditions: measured by three persons or less per sleeping room
4. Being connected to outside support: measured by household ownership of at least one phone (landline or mobile)
5. Access to up-to-date information about the pandemics: measured by mass-media exposure among caregivers

Confidential: For Review Only

We declare no competing interests.

Funding sources: UKRI GCRF Collective Fund Award (Grant Ref: ES/T003936/1) to the University of Oxford, UKRI ECRS GCRF, Harnessing the power of global data to support young children's learning and development: Analyses, dissemination and implementation.

References

1. UN General Assembly, Convention on the Rights of the Child, 20 November1989,United Nations, Treaty Series,Vol.1577,p3. https://www.ohchr.org/en/instruments-mechanisms/instruments/convention-rights-child
2. Moon, S., Armstrong, J., Hutler, B., Upshur, R., Katz, R., Atuire, C., ... & Wolff, J. Governing the access to COVID-19 tools accelerator: towards greater participation, transparency, and accountability. *The Lancet*. 2022 Jan 29;399(10323):487-494. doi: 10.1016/S0140-6736(21)02344-8.
3. World Health Organization. Coronavirus disease (COVID-19): Home care for families and caregivers. https://www.who.int/news-room/q-a-detail/coronavirus-disease-covid-19-home-care-for-families-and-caregivers. 2020 (Geneva: World Health Organization). Accessed 3 May 2021.
4. United States Central for Disease Control and Prevention (CDC). CDC Museum COVID-Timeline. https://www.cdc.gov/museum/timeline/covid19.html#:~:text=December%2012%2C%202019,shortness%20of%20breath%20and%20fever (accessed June 6, 2022)
5. Lu C, Luan Y, Naicker SN, Subramanian SV, Behrman JR, Heymann J, Stein A, Richter LM. Assessing the prevalence of young children living in households prepared for COVID-19 in 56 low- and middle-income countries. Glob Health Res Policy. 2022 Jun 21;7(1):18. doi: 10.1186/s41256-022-00254-2. PMID: 35729611; PMCID:

PMC9210057.

6. Overcrowded households. 2018; (accessed April 26, 2020). https://www.ethnicity-facts-figures.service.gov.uk/housing/housing-conditions/overcrowded-households/latest
7. Progress on household drinking water, sanitation and hygiene 2000-2017. Special focus on inequalities. New York: United Nations Children's Fund (UNICEF) and World Health Organization, 2019. https://www.unicef.org/reports/progress-on-drinking-water-sanitation-and-hygiene-2019
8. O'Hare BAM, Lopez MJ, Mazimbe B, Murray S, Spencer N, Torrie C, et al. (2022) Tax abuse—The potential for the Sustainable Development Goals. PLOS Glob Public Health 2(2):e0000119. https://doi.org/10.1371/journal.pgph.0000119
9. Strong systems and sound investments: evidence on and key insights into accelerating progress on sanitation, drinking-water and hygiene. The UN-Water global analysis and assessment of sanitation and drinking-water (GLAAS) 2022 report. Geneva: World Health Organization; 2022. https://www.unwater.org/publications/un-water-glaas-2022-strong-systems-and-sound-investments-evidence-and-key-insights

Confidential: For Review Only
